# Relevance of MicroRNA200 Family and MicroRNA205 for Epithelial to Mesenchymal Transition and Clinical Outcome in Biliary Tract Cancer Patients

**DOI:** 10.3390/ijms17122053

**Published:** 2016-12-07

**Authors:** Romana Urbas, Christian Mayr, Eckhard Klieser, Julia Fuereder, Doris Bach, Stefan Stättner, Florian Primavesi, Tarkan Jaeger, Stefanie Stanzer, Anna Lena Ress, Magdalena Löffelberger, Andrej Wagner, Frieder Berr, Markus Ritter, Martin Pichler, Daniel Neureiter, Tobias Kiesslich

**Affiliations:** 1 Institute of Pathology, Paracelsus Medical University/Salzburger Landeskliniken (SALK), Salzburg 5020, Austria; r.urbas@salk.at (R.U.); e.klieser@salk.at (E.K.); 2 Laboratory for Tumour Biology and Experimental Therapies (TREAT), Institute of Physiology and Pathophysiology, Paracelsus Medical University, Salzburg 5020, Austria; christian.mayr@pmu.ac.at (C.M.); mloeffelberger.bma-b2012@fh-salzburg.ac.at (M.L.); frieder.berr@pmu.ac.at (F.B.); markus.ritter@pmu.ac.at (M.R.); t.kiesslich@salk.at (T.K.); 3 Department of Internal Medicine I, Paracelsus Medical University/Salzburger Landeskliniken (SALK), Salzburg 5020, Austria; j.fuereder@gmx.net (J.F.); doris.bach@gmx.at (D.B.); and.wagner@salk.at (A.W.); 4 Department of Visceral-, Transplant- and Thoracic Surgery, Medical University of Innsbruck, Innsbruck 6020, Austria; stefan.staettner@tirol-kliniken.at (S.S.); florian.primavesi@tirol-kliniken.at (F.P.); 5 Department of Surgery, Paracelsus Medical University/Salzburger Landeskliniken (SALK), Salzburg 5020, Austria; ta.jaeger@salk.at; 6 Research Unit for Non-Coding RNA and Genome Editing in Cancer, Division of Oncology, Department of Internal Medicine, Medical University of Graz, Graz 8036, Austria; Stefanie.Stanzer@klinikum-graz.at (S.S.); anna.lena.ress@gmail.com (A.L.R.); martin.pichler@medunigraz.at (M.P.); 7 Department for Radon Therapy Research, Ludwig Boltzmann Cluster for Arthritis and Rehabilitation, Salzburg 5020, Austria

**Keywords:** biliary tract cancer, epithelial to mesenchymal transition, microRNA-200 family, microRNA-205

## Abstract

Extensive stromal interaction is one reason for the dismal outcome of biliary tract cancer (BTC) patients. Epithelial to mesenchymal transition (EMT) is involved in tumor invasion and metastasis and is partly regulated by microRNAs (miRs). This study explores the expression of anti-EMT miR200 family (miR141, −200a/b/c, −429) and miR205 as well as the EMT-related proteins *E*-cadherin and vimentin in a panel of BTC cell lines and clinical specimens by quantitative real-time polymerase chain reaction, Western blot and immunohistochemistry, respectively. MicroRNA expression was correlated to (i) the expression patterns of *E*-cadherin and vimentin; (ii) clinicopathological characteristics; and (iii) survival data. MicroRNA-200 family and miR205 were expressed in all BTC cells and clinical specimens. *E*-cadherin and vimentin showed a mutually exclusive expression pattern in both, in vitro and in vivo. Expression of miR200 family members positively correlated with *E*-cadherin and negatively with vimentin expression in BTC cells and specimens. High expression of miR200 family members (but not miR205) and *E*-cadherin was associated with longer survival, while low miR200 family and high vimentin expression was a predictor of unfavorable survival. Overall, the current study demonstrates the relevance of the miR200 family in EMT of BTC tumors and suggests these miRs as predictors for positive outcome.

## 1. Introduction

MicroRNAs (miRs) are small non protein-coding RNA molecules with a length of about 21–25 nucleotides that play a key role in the regulation of gene expression. Via complementary base pairing with protein-coding RNA species (mRNAs), miRs cause either degradation (perfect match) or prevention of translation (imperfect match) of the respective mRNAs [1]. At present, almost 30,000 miR species are known [2], and it is clear today that aberrant miR expression plays a crucial role in development and progression of various types of cancers [3,4].

Epithelial to mesenchymal transition (EMT) is a key process in the progression of cancer, in which epithelial cells lose their polarity and gain mesenchymal traits, including the ability to detach from the primary tumor, invade surrounding tissue and eventually form metastases [5,6,7]. The miR200 family (miR141, 200a, 200b, 200c and 429) as well as miR205 were shown to negatively regulate EMT [6,8]. Two studies demonstrated that the miR200 family directly targets the EMT-promoting transcription factors ZEB1 and ZEB2, repressors of the epithelial marker and anti-EMT factor *E*-cadherin. As a consequence, expression of *E*-cadherin was increased, leading to reduction of the invasive potential of cancer cells and making miR200 a potent tumor suppressor [6,9].

Biliary tract cancer (BTC) is a malignant disease of biliary tract epithelial cells that arises at different localizations within the biliary tree system, including the gall bladder [10,11]. Although BTC is generally rare in Western countries, it is the second most common hepatic malignancy after hepatocellular carcinoma [12]. While knowledge about BTC tumorigenesis is increasing, the prognosis and survival of patients with BTC is still very poor—mainly because of late diagnosis, when cancer has spread and is no longer resectable by surgery, and due to lack of effective therapeutic options and second line therapies. This, together with high resistance to common chemotherapies, leaves only palliative treatment for most patients with a median survival of about one year [13,14].

EMT is a known mechanism of BTC cells to invade surrounding tissue and to form metastases, contributing to the aggressive nature of these tumors [15]. In previous studies, expression of miR200 family members was found to be downregulated in primary sclerosing cholangitis and BTC cells/specimens compared to normal tissue, leading to EMT activation and enhancement of invasive potential [16,17,18,19]. In addition, Peng et al. demonstrated in 2013 that low expression of miR200 b and c resulted in formation of large tumors in a BTC mouse model as well as in enhanced formation of tumor spheres and expression of the cancer stem cell surface marker CD133 [17]. Conversely, ectopic expression of miR200 family members in BTC cells reduced sphere formation, expression of CD133 as well as invasion rate, formation of metastases and triggering of EMT in vitro and in vivo, underlining the crucial role of the miR200 family, especially regarding EMT in BTC [16,17]. However, in contrast to these findings, over-expression of miR141 was found to be a predictor of poor prognosis and shorter disease-free and overall survival in another study, demonstrating that members of the miR200 family may act as both oncogenes and tumor suppressors in BTC [20].

The aim of the present study was to investigate the role of the miR200 family as well as miR205 in a preclinical model and a BTC patient cohort for induction of EMT, using the established EMT markers *E*-cadherin and vimentin.

## 2. Results

### 2.1. Expression of miR200 Family Members, miR205 and Epithelial to Mesenchymal Transition (EMT) Markers in Biliary Tract Cancer (BTC) Cell Lines

**Figure 1:**
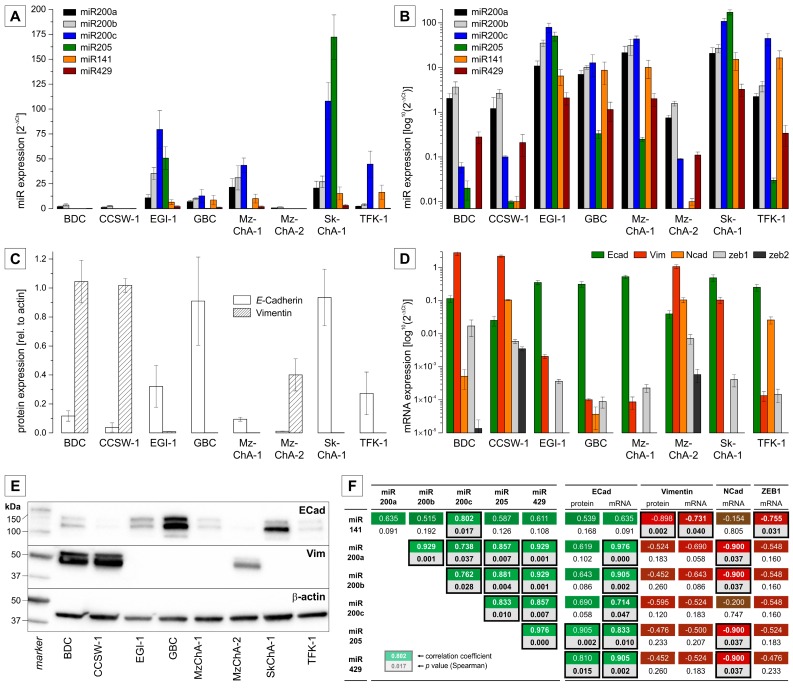
Relative expression of miR200 family members, miR205 and EMT-related markers (mRNA, protein) in BTC cell lines. (**A**,**B**) expression levels of miRs related to RNU6B using a linear (**A**) and a logarithmic (**B**) scale, respectively; (**C**) quantitative protein expression (densitometry) of epithelial marker *E*-cadherin and mesenchymal marker vimentin; (**D**) mRNA levels of EMT markers related to β-actin; (**E**) representative Western blot images (cropped); (**F**) Spearman correlation analysis of miR expression with miR expression as well as mRNA/protein expression of EMT markers (*E*-cadherin, vimentin, *N*-cadherin, ZEB1). The first numeric value in each analysis (rectangle) represents the correlation-coefficient (**green** for positive correlation, **red** for negative correlation), the second represents the *p*-value (highlighted **grey** when significant). Rectangles of significant correlations are highlighted by a **black** border. Abbreviations: BTC: biliary tract cancer; Ecad: *E*-cadherin; EMT: epithelial to mesenchymal transition; miR: MicroRNA; Vim: Vimentin.

In an in vitro system of a panel of BTC cell lines (*n* = 8), expression of members of the miR200 family and miR205 could be detected in all cell lines, with the EGI-1, GBC, MzChA-1, SkChA-1 and TFK-1 cell lines showing relatively high miR expression and the BDC, CCSW-1 and MzChA-2 cell lines showing comparably lower expression (Figure 1A,B).

On the protein level, we measured the expression of *E*-cadherin and Vimentin as the most important markers of an epithelial and mesenchymal phenotype, respectively. As shown in Figure 1C, cell lines with low miR200 family and low miR205 expression (BDC, CCSW-1 and MzChA-2) had low or undetectable *E*-cadherin and high vimentin expression. In contrast, the cell lines EGI-1, GBC, MzChA-1, SkChA-1 and TFK-1 are characterized by (high) E-cadherin and low or undetectable vimentin, and, accordingly, higher miR200 family and miR205 expression. Similar results were obtained on the mRNA level for both of these markers (Figure 1D). Additionally, we measured the mRNA expression levels of pro-oncogenic and pro-metastatic *N*-cadherin, which is thought to replace *E*-cadherin in a process referred to as “the cadherin switch” during EMT [21,22], as well as of EMT transcription factors ZEB1/2 [23] and found expression patterns that are similar to that of vimentin (Figure 1D).

A correlation analysis between miR200 family members and EMT markers *E*-cadherin, vimentin, *N*-cadherin and ZEB1 is shown in Figure 1F. With the exception of miR141, all tested miRs show a significant positive correlation with *E*-cadherin mRNA expression. For *E*-cadherin protein levels, miR205 and miR429 show a significant positive correlation; similarly, for the other miRs, correlation analysis versus *E*-Cadherin reveals a clear positive trend. As expected, a negative correlation was observed between the expression of miR200 family members/miR205 and EMT-related factors vimentin (mRNA and protein), *N*-cadherin and ZEB1 (both mRNA). Of note, a positive and significant correlation of miR200 family members, and miR205, respectively, with each other was observable in the set of eight BTC cell lines.

### 2.2. Expression of miR200 Family Members and EMT Markers in Clinical BTC Cases

MicroRNAs were isolated from archived formalin-fixed paraffin-embedded (FFPE) material (*n* = 78 BTC cases, summarized in Table 1) and measured by quantitative real-time polymerase chain reaction (PCR). The tumor localization was mainly intrahepatic (50%), followed by perihilar (28%), within the gall bladder (13%), and extrahepatic (9%). Only classification by basic clinicopathological data, such as tumor grading, tumor invasion, and lymph node metastasis according to the TNM Classification of Malignant Tumors (TNM) and Union for International Cancer Control (UICC) classification [24] resulted in significant different distributions between the four localizations. All other variables, such as median age or gender, showed no significant different distributions (Table 1).

**Figure 2:**
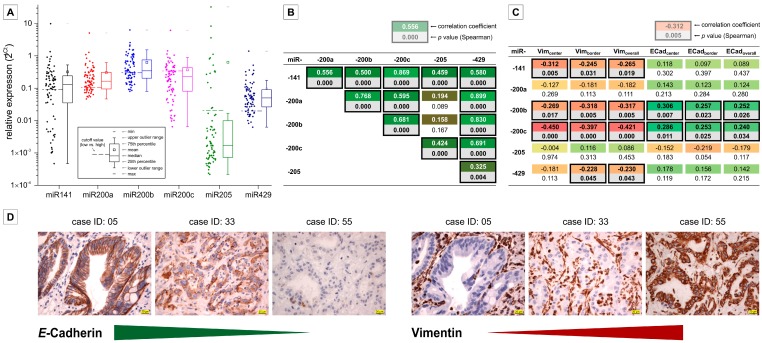
Expression of miR200 family members, miR205 and EMT markers *E*-cadherin and vimentin in clinical BTC samples. (**A**) Relative expression (to reference miRs RNU6 and SNORD61) of miR200 family members and miR205 in *n* = 78 BTC cases (formalin-fixed paraffin-embedded material); (**B**) Spearman correlation analysis of miR expression levels in BTC cases. The first numeric value in each analysis (rectangle) represents the correlation-coefficient (ranging from **green** for positive correlation to **red** for negative correlation), the second represents the *p*-value (highlighted **grey** when significant). Significant correlations are highlighted by a **black** border; (**C**) Spearman correlation analysis of miR expression levels and vimentin, respectively, and *E*-cadherin expression patterns in BTC cases; and (**D**) representative immunohistochemistry images of three BTC cases exemplifying inverse expression patterns of *E*-cadherin and vimentin. Scale bar (**yellow**) indicates 20 µm. Abbreviations: BTC: Biliary tract cancer; Ecad: *E*-Cadherin; EMT: Epithelial to mesenchymal transition; miR: MicroRNA; Vim: Vimentin.

As summarized in Figure 2A, the five analyzed miR200 members as well as miR205 were detectable in all clinical BTC samples. MicroRNA-141 and miR205 were more heterogeneously expressed compared to the remaining miRs, which showed a smaller range of expression. It is worth noting that miR205 generally showed the lowest expression in the analyzed patient samples. Similar to the results obtained from the in vitro cell line experiments, the expression of each miR200 family member significantly positively correlates with the expression of each other tested miR (Figure 2B). In addition, we measured expression levels of E-cadherin and vimentin in all clinical BTC samples via semi-quantitative immunohistochemistry. Figure 2D illustrates examples of cases with low, medium and high expression, respectively, for each of the two proteins. Correlation analysis proves a highly significant inverse correlation between vimentin and *E*-cadherin (Spearman′s correlation coefficient = −0.729, *p* < 0.001). In accordance with the data obtained by the in vitro experiments, the expression of these proteins is nearly mutually exclusive, i.e., high *E*-cadherin is associated with low vimentin expression and vice versa. Comparing those data to the case-specific expression levels of miR200 family members, a positive correlation of miR expression with expression of *E*-cadherin, and a negative correlation with expression of vimentin (Figure 2C) was observed. Of note, these calculations were highly significant for miR200b and miR200c. In contrast, miR205 expression—by trend—negatively correlated with *E*-cadherin and (for border and overall expression) correlated positively with vimentin expression.

### 2.3. Association with Clinicopathological Parameters

**Figure 3:**
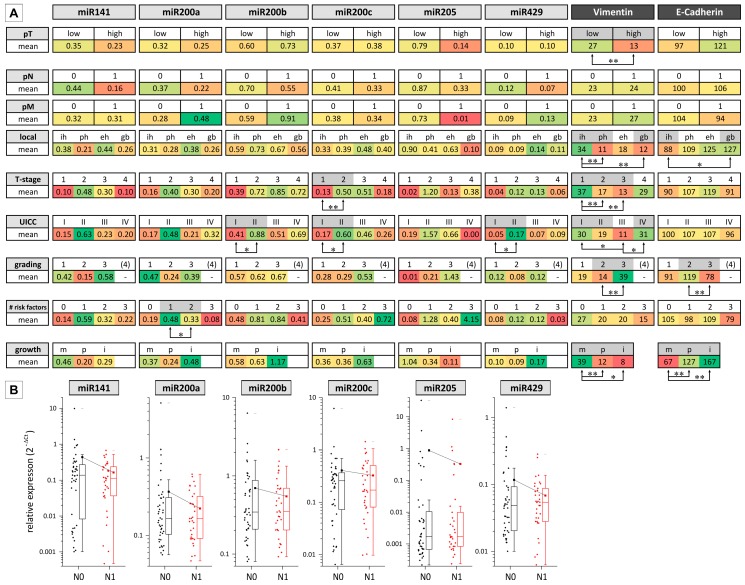
Association of miR200 family/miR205 expression with clinicopathological parameters for *n* = 78 biliary tract cancer cases. (**A**) For each variable (miRs (qRT-PCR), vimentin, *E*-cadherin (semi-quantitative immunohistochemistry)), the mean expression value is shown and highlighted by color (ranging from green = high expression to red = low expression). Differences between grading could be analyzed only for G1–G3 as the cohort included only one case of G4. Significant differences between the two group or more groups (calculated using a Student′s *t*-test and ANOVA/LSD (least significant difference) *post hoc* test, respectively) are highlighted by grey shading and arrows (* *p* < 0.05 and ** *p* < 0.01, respectively); (**B**) microRNA expression pattern exemplified for N0 and N1 groups of patient samples. Abbreviations: eh, extrahepatic; gb, gall bladder; i, intraductal; ih, intrahepatic; m, mass-forming; p, periductal; ph, perihilar.

Next, we related expression of miR200 family members and miR205 to the following clinicopathological parameters using a Student′s *t*-test, ANOVA and post hoc analysis (Figure 3): T-stage (1–4), N-status (0–1), M-status (0–1), UICC (1–4), grading (1–4), number of risk factors (0–3), localization (intrahepatic, extrahepatic, perihilar, gallbladder), and growth pattern (intraductal, periductal and mass-forming).

Regarding vimentin and *E*-cadherin, we noticed a significant difference in overall expression for grade 2 and grade 3 tumors: G1 and G2 tumors displayed lower vimentin and higher *E*-cadherin expression, whereas G3 tumors displayed higher vimentin and lower *E*-cadherin expression. Significant different expression patterns of vimentin and *E*-cadherin were also seen regarding the different localizations of BTC and the T-stage. For vimentin, the expression was highest in intrahepatic tumors, whereas in perihilar and gallbladder tumors, the expression was lowest (*p* < 0.05). *E*-cadherin expression, on the other hand, was lowest in intrahepatic tumors and highest in gallbladder cancer samples (*p* < 0.05). In addition, we observed significant lower vimentin expression in UICC III versus UICC I cases. Interestingly, between UICC III and IV, the expression markedly increased (*p* < 0.05).

Expression of miRs was higher in patients categorized as T-stage 2 compared to stage 1 (significant for miR200c). Similarly, regarding UICC classification, miRs showed an increase in expression in tumors categorized as UICC II compared to UICC I (significant for miR200b, −200c, and −429). Analysis of miR expression regarding lymphatic metastasis revealed a trend towards reduced expression of all miR species in N1 tumors versus N0 (not significant; see Figure 3B for details), whereas the classification of cases regarding pT stage and distant metastases (M0/1) was not associated with a significant different miR expression. Furthermore, classification of tumor samples according to the growth pattern, grading, localization, and number of risk factors did not reveal considerable miR expression differences. In contrast, vimentin/E-cadherin expression decreased/increased significantly from mass-forming, periductal to intraductal growth pattern, respectively.

### 2.4. Association of miRNA-200 Family Members and Overall Survival

**Figure 4:**
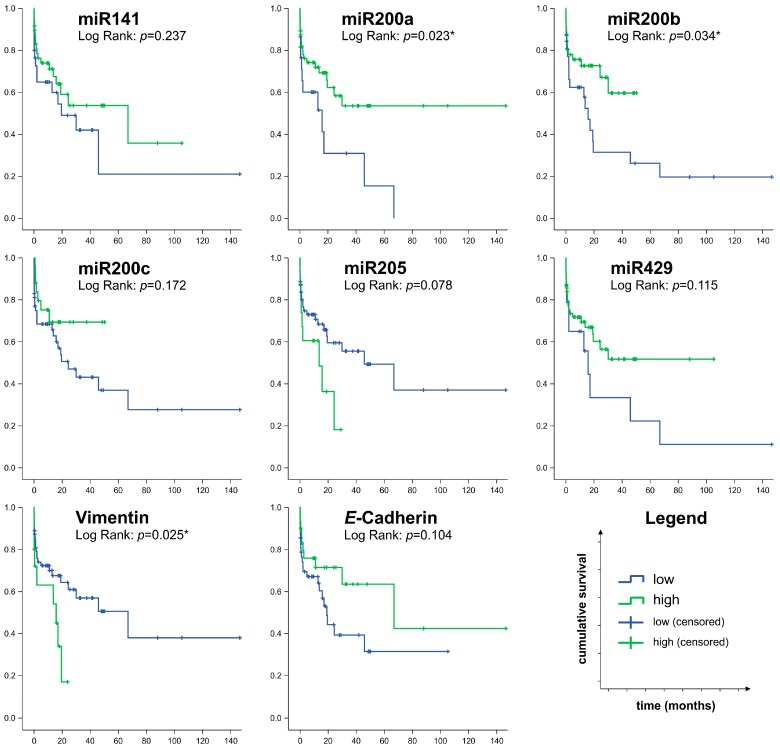
Survival analysis of biliary tract cancer (*n* = 78) cases with regard to expression levels of miR200 family members, miR205, *E*-cadherin and vimentin. The cut-off value for distinguishing cases with low or high expression of the respective miRNA or protein was determined by the receiver operating characteristic (ROC) calculation and Youden Index analysis.

Finally, we performed survival analysis using the receiver operating characteristic (ROC) calculation and Youden Index analysis to determine miR-specific cut-off values (shown in Figure 2A) to categorize the tumor samples into high and low miR-expressing BTC cases (Figure 4).

Uniformly, high expression of miR200 family (−141, −200a/b/c, −429) members was associated with prolonged overall survival compared to cases expressing low levels of these miRs: using the log rank test, statistically significant differences could be observed for miR200a and miR200b. In contrast, classification of cases by low versus high miR205 expression indicated that high expression was associated with shorter overall survival. As expected, high expression of epithelial *E*-cadherin was a predictor of longer survival (*p* = 0.104), while high expression of the mesenchymal marker vimentin was present in patients with short survival compared to cases with low vimentin expression (*p* = 0.025).

In line with these results, Cox regression analysis (Table 2) identified metastatic spread (pM) and miR205 expression as significant negative predictors of survival (hazard ratio 3.68 and 3.40, respectively; *p* < 0.05). When using the backward Wald method for Cox regression analysis, pM (hazard ratio (*HR*) = 3.00, *p* = 0.007), miR200b (*HR* = 0.37, *p* = 0.009) and miR205 (*HR* = 2.44, *p* = 0.031) remained as relevant variables in the model.

## 3. Discussion

In the present study, we evaluated the relevance of miR200 family members and miR205 for the EMT phenotype in BTC in vitro and in vivo. In initial reports, it became apparent that the miR200 family members (miR141, −200a/b/c, −429) acted as anti-EMT miRs in BTC [16,17].

In a comprehensive BTC cell model, the five miR200 family members were quantifiable in all tested cell lines and their expression positively correlated with the expression of *E*-cadherin and negatively with the expression of vimentin, *N*-cadherin and ZEB1. These expression patterns are consistent with the finding that miR200 members directly target the EMT-promoting transcription factor ZEB1, which itself is an inhibitor of the epithelial marker *E*-cadherin [23,25]. The expression patterns we measured within the present study prove that high expression of miR200 family members is a potential marker for *E*-cadherin-positive BTC cells, since only cell lines with high expression of these miRs also expressed *E*-cadherin at a considerable amount—whereas vimentin as a mesenchymal marker is present only in cell lines expressing low miR200 levels. This observation goes in line with Park et al., who showed that miR200 is exclusively expressed in *E*-cadherin-positive cancer cells of different origin [6]. Additionally, Sundararajan and coworkers recently showed, in a cell line panel representing nine different cancer entities, that miR200c expression was high only in epithelial cells, whereas ZEB1 expression was high in mesenchymal cell types [26].

In BTC specimens, we found a similar correlation between expression of miR200 family members and high *E*-cadherin and low vimentin levels. For miR200b and 200c, this observation was most evident (highly significant). In primary ovarian cancer specimens, expression of miR200 resulted in upregulation of *E*-cadherin, confirming the relationship between the miR200 family and *E*-cadherin [6]. Additionally, the analysis of both a BTC cancer cell line panel and clinical BTC samples revealed a significant positive correlation of the expression patterns of the miR200 family members, thus indicating a coordinated expression of these miRs, which has also been postulated earlier [27].

MicroRNA205 is another miR species that was shown to negatively regulate EMT [8]. For example, in a recent study on gastric cancer, miR205 suppressed the invasive potential of gastric cancer cells [28]. Furthermore, the authors confirmed that ZEB1 is a direct target of miR205 and that artificial upregulation of miR205 resulted in enhanced *E*-cadherin (probably by downregulation of ZEB1) and diminished *N*-cadherin and vimentin levels [28]. However, in our study, miR205 expression negatively correlated with *E*-cadherin, and, in part, correlated positively with vimentin expression, probably suggesting that miR205 may act as a supporter of EMT rather than a suppressor. In addition—and in contrast to the data obtained for miR200 family members—high expression of miR205 correlated with poor overall survival.

This result reflects the controversial role of miR205 regarding EMT as described in the current literature: Gregory et al. recognized downregulation of miR205 in cells that had undergone EMT, suggesting that miR205 acts as an anti-EMT miR in this context. Of note, this downregulation was accompanied by downregulation of all five miR200 family members, indicating a functional connection of miR200 family and miR205 [8]. In another study, regarding prostate cancer, the authors compared metastatic versus non-metastatic prostate cancer xenograft samples. While they found high miR205 expression in non-metastatic tissue, miR205 was absent in metastatic tissue [29]. Furthermore, over-expression of miR205 resulted in reduced invasiveness and metastatic potential in prostate and colon cancer cells [30,31]. Regarding BTC, a study by Okamoto et al. demonstrated that over-expression of miR205 enhanced sensitivity to the standard chemotherapeutic Gemcitabine, which, again, categorizes miR205 as a tumor suppressor miR in this particular case [32]. In contrast, there are studies that describe miR205 as an oncogenic miR, and, in line with our results, connect miR205 expression with increased metastatic potential and poor outcomes. In a work conducted by Kalogirou et al., high miR205 resulted in higher cancer-specific mortality [33]. In ovarian cancer, upregulated miR205 expression was associated with advanced pathological grade and clinical stage [34]. Interestingly, in the same study, artificial over-expression of miR205 resulted in increased motility. Likewise, in nasopharyngeal carcinoma cells, enhanced miR205 expression increased proliferation, invasion and migration [35]. In oral cavity and oropharynx squamous cell carcinoma, enhanced miR205 expression correlated with disadvantageous clinicopathological parameters, including vascular invasion and lymph node metastasis [36]. The results obtained in the present study, suggesting that miR205 acts as an oncogenic miR and a predictor of poor prognosis, combined with the controversial data obtained from the literature, underline the dual role of miR205 in cancer and may indicate oncogenic roles of this miR in BTC. Identifying additional targets of miR205 and investigating the probably tumor-specific importance of individual targets might explain this phenomenon.

Besides their role in EMT and metastasis, the miR200 family might be involved in other aspects of BTC progression, and, in this regard, they might be both tumor suppressors and oncogenic miRs. As shown by Wiklund et al. [27] in bladder cancer, expression of miR200 family members and miR205 was enhanced in tumor samples compared to healthy control samples. However, expression of these miRs was decreased in invasive tumors, suggesting that miR200 family and miR205 intrinsically have oncogenic potential but also inhibit tumor invasion and metastasis [27]. Regarding BTC, in a recent study by Goeppert and coworkers, miR200a, miR200b and miR429 were found to be upregulated in BTC specimens compared to control samples [37]. Furthermore, the authors demonstrated that the potential BTC-related tumor suppressor CDH6 is a direct target of miR429, and that downregulation of CDH6 occurs stepwise during BTC progression (with BTC showing the lowest levels) and is associated with poor overall survival [37]. The Polycomb Repressive Complexes (PRC) are epigenetic regulators that are overly active in cancer cells and are involved in development and progression of BTC [38,39,40]. Peng et al. identified SUZ12, a core component of the epigenetic regulator Polycomb Repressive Complex 2 (PRC2), as a direct target of miR200b and 200c [17]. Interestingly, BMI1, a core component of the PRC1, was suggested to be a direct target of miR429 in another study. Moreover, they demonstrated that downregulation of miR429 not only reduced the expression of *E*-cadherin, but also the expression of the key tumor suppressors p14 and p16. Therefore, future studies should not only give more detailed information about the role of miR200 family members and miR205 in EMT, but should also regard other aspects of tumorigenesis and progression.

Overall survival is probably the most important endpoint, and, for this parameter, several studies indicate that miR200 family expression correlates with better outcome. In bladder cancer, high miR200c expression was a predictor of prolonged survival time [27]. Regarding BTC, Oishi and coworkers observed that high expression of miR200c was associated with longer overall and recurrence-free survival [16]. In line with these results, high expression of miR200c resulted in better cumulative survival in our BTC cohort compared to cases with low miR200c expression. In addition, high expression of the remaining four members of the miR200 family was similarly associated with longer overall survival (significant for miR200b).

## 4. Materials and Methods

### 4.1. Cell Culture and Clinical BTC Samples

Biliary tract cancer cell lines used in this study ([11], see [39] for references) included five bile duct carcinoma (BDC (G4), CCSW-1 (G2), EGI-1 (G3), SkChA-1 (G3), and TFK-1 (G2)) and three gallbladder carcinoma cell lines (GBC (G1), MzChA-1 (G1), and MzChA-2 (G2)) which were cultured in Dulbecco′s modified Eagle′s Medium supplemented with 10% (*v*/*v*) fetal bovine serum (FBS; Gibco, Life Technologies, Waltham, MA, USA) as described before [39,41,42]. For miRNA, mRNA and protein expression analysis, cells were seeded in 60 mm petri dishes 24 h prior to RNA isolation at 75% confluency. Seventy-eight cases of FFPE BTC samples archived between 1997 and 2015 at the Institute of Pathology (Paracelsus Medical University, Salzburg, Austria) were included in the current study. All analyses on human BTC samples were carried out on routinely archived and anonymized specimens according to the local ethics committee (Reference No. 415-EP/73/37-2011). The clinical and pathologic characteristics are summarized in Table 1.

### 4.2. MicroRNA, mRNA and Protein Expression Analysis from BTC Cell Lines

Total RNA was isolated using Trizol reagent according to the manufacturer′s instructions (Invitrogen/Thermo Fisher, Waltham, MA, USA), quantified by photometry (260 nm, U-2000 Spectrophotometer, Hitachi, Wr. Neudorf; Austria), transcribed for miRNA expression analysis using the TaqMan MicroRNA Reverse Transcription Kit (Applied Biosystems/Thermo Fisher, Waltham, MA, USA) and analyzed for miR200 family members miR141, 200a, 200b, 200c and 429 and miR205 expression using TaqMan miRNA Assays and the TaqMan Universal Master Mix II on a ViiA7 real-time PCR thermocycler (ABI/Thermo Fisher) according to the manufacturer′s instructions. Results obtained from three independent biological replicates were normalized to the RNU6B housekeeping miRNA.

For mRNA expression analyses, total RNA was treated with RQ RNase-free DNase, transcribed using the ImProm-II Reverse Transcription System and random hexamer primers and analyzed using GoTaq Green Master Mix (all from Promega, Mannheim, Germany, according to the manufacturer′s instructions) and custom-designed primers (Sigma-Aldrich, Vienna, Austria). Results obtained from three independent biological replicates were normalized to the β-actin housekeeping gene. Primer sequences are available from the corresponding author upon request.

For Western blot analysis, at 24 h post seeding, cells were harvested, counted and the pellet was frozen at −20 °C until further processing. Samples of each replicate were thawed, resuspended in an appropriate volume of phosphate buffered saline (PBS) to obtain approximately 30 µg total cell protein per 10 µL solution (1.10^5^ cells/10 µL), sonicated 8 times (total 10 s; Sonopuls HD70, Bandelin, Berlin, Germany), mixed with one volume of sodium dodecyl sulfate (SDS) sample buffer (0.125 M Tris (pH 6.8), 4% SDS, 20% Glycerol, 0.02% bromophenol blue, 200 mM dithiothreitol (DTT)), heated for 5 min at 95 °C and separated on a gradient SDS gel (4%–20% Mini-PROTEAN^®^ TGX Stain-Free, Biorad, Munich, Germany). Proteins were transferred by Trans-Blot Turbo to nitrocellulose membranes (Biorad) and—after standard washing and blocking procedures (nonfat dry milk blocking buffer)—probed for *E*-cadherin (#3195, 1:1000), vimentin (#5741, 1:1000) and β-actin (#8457, 1:1000; all purchased from Cell Signaling Technologies, Danvers, MA, USA) and detected by SignalFire™ enhanced chemiluminescence (ECL) Reagent (Cell Signaling Technologies) on a ChemiDoc MP System (Biorad). Band intensities were quantified using ImageJ (version 1.48v; available online: imagej.nih.gov) and, after blot-internal calibration to the respective most intensive band, related to the corresponding intensity of the β-actin bands.

### 4.3. Expression Analysis of miR200 Family and EMT Protein Markers in FFPE Samples

Depending on the size of the tumor area on the FFPE block, three to ten 10 µm sections comprising the tumor tissue without surrounding tissue were cut using a RM2245 semi-automated rotator microtome (Leica Biosystems Nussloch GmbH, Nussloch, Germany), transferred to sterile 1.5 mL tubes and further processed using the miRNeasy FFPE Kit according to the manufacturer’s instructions. MicroRNAs were transcribed using the miScript II RT Kit and quantified on a ViiA7 thermocycler using QuantiTect SYBR Green mastermix and miScript Primer Assays. Using the DataAssist software (version 3.01; Applied Biosystems/Thermo Fisher), all results were normalized to the expression of RNU6 and SNORD61 as reference miRs. All FFPE miR reagents and qPCR kits were obtained from Qiagen (Hilden, Germany) and used according to the manufacturer’s instructions.

*E*-Cadherin and vimentin as the most important epithelial and mesenchymal marker proteins, respectively, were measured by semi-quantitative immunohistochemistry on all FFPE tissue specimens. In brief, 4 µm sections were mounted on glass slides, deparaffinized using graded alcohols, subjected to antigen retrieval at pH9 and stained using primary antibodies (mouse-monoclonal anti-*E*-cadherin (36, catalogue No. 790-4497) and anti-vimentin (V9, catalogue No. 790-2917), each ready-to use; Ventana Medical Systems, Basel, Switzerland) on a Benchmark Ultra (Ventana). Staining intensity (0–3) and extent (0%–100% tumor cells) were assessed by experienced pathologists (RU, EK, DN) for the tumor center and borders. Semi-quantitative immunohistochemistry scores were calculated by multiplication of the intensity and extensity yielding scores between 0 and 300 [43].

### 4.4. Statistics

All calculations were performed using IBM SPPS Statistics 20 (IBM Austria, Vienna, Austria). Statistical results were considered significant (*) or highly significant (**) at *p* < 0.05 and *p* < 0.01, respectively. Correlation analysis (miRs versus miRs, mRNAs, and protein) were performed using Spearman’s correlation analysis. Differences in the clinicopathological data for each localization (intrahepatic, extrahepatic, perihilar, gall bladder) were analyzed using the χ^2^ test and ANOVA (Bonferroni *post hoc* test) for nominal and continuous variables, respectively. Differences in miRNA and vimentin/*E*-cadherin expression between patient samples grouped by clinicopathological parameters were calculated using a Student′s *t*-test and ANOVA and LSD *post hoc* tests. Cut-off values for miRs in our FFPE sample cohort were determined using the receiver operating characteristic (ROC) calculation and Youden Index analysis for overall survival. Survival curves were generated using the Kaplan–Meier method and compared by log rank tests (Mantel–Cox). Cox regression analysis to identify predictors of survival were performed using either the enter or backward elimination Wald method.

## 5. Conclusions

In summary, this study clearly confirms an association between miR200 family members and the BTC cellular phenotype in vitro and in vivo. The expression of these miRNAs is significantly associated with an epithelial phenotype and might serve as a positive predictor of overall survival in BTC patients. Subsequent mechanistic studies, based on over-expression or inhibition of specific miRNAs, have to prove the functional connection between EMT-related changes in cellular phenotype (including migration, invasion capabilities) and the individual role of miR200 family members in BTC.

## Figures and Tables

**Table 1:** Clinical and pathological characteristics of biliary tract cancer (BTC) cases.

*n* Samples		Localization					All BTC Samples
		Intrahepatic	Perihilar	Extrahepatic	Gall Bladder	χ^2^ Test/ANOVA	
*n* (%)		39 (50.0%)	22 (28.2%)	7 (9.0%)	10 (12.8%)		78
Female (%)		14 (38.9%)	11 (30.6%)	3 (8.3%)	8 (22.2%)	*p* = 0.093 (χ^2^)	36 (46.2%)
Male (%)		25 (59.5%)	11 (26.2%)	4 (9.5%)	2 (4.8%)		42 (53.8%)
Age: mean (95% CI, years)		65.9 (62.2–69.7)	69.4 (65.7–73.1)	72.0 (60.8–83.2)	70.1 (61.4–78.9)	*p* = 0.379 (ANOVA)	68.0 (65.6–70.4)
Growth pattern ^a^: m/p/i		28/11/0	3/18/1	1/5/1	2/7/1	*p* < 0.001 ** (χ^2^)	34/41/3
Grading: 1/2/3/4		4/19/16/0	0/18/4/0	0/4/2/1	0/4/6/0	*p* = 0.011 * (χ^2^)	4/45/28/1
Size: mean (95% CI, cm)		5.3 (4.0–6.6) ^#^	2.5 (1.5–3.6) ^#^	2.5 (1.2–3.8)	2.4 (1.5–3.6)	*p* = 0.004 ** (ANOVA)	3.9 (3.1–4.7)
T staging	1/1b	21/0	1/0	0/0	2/1	*p* < 0.001 ** (χ^2^)	24/1
	2/2a/2b	1/7/4	2/4/14	2/0/0	2/3/0		7/14/18
	3	4	0	5	2		11
	4	2	1	0	0		3
N status ^b^: 0/1		23/16	10/12	3/4	8/2	*p* = 0.265 (χ^2^)	44/34
M status ^b^: 0/1		35/4	17/5	6/1	10/0	*p* = 0.302 (χ^2^)	68/10
UICC	I/II/III/IV	16/9/1/13	2/7/9/4	2/4/0/1	3/3/4/0	*p* = 0.001 ** (χ^2^)	23/23/14/18
R status ^b^: 0/1		27/12	17/5	6/1	7/3	*p* = 0.776 (χ^2^)	57/21
Etiology: known/unknown		17/22	15/7	3/4	3/7	*p* = 0.155 (χ^2^)	38/40
Survival: no/yes		21/18	12/10	5/2	7/3	*p* = 0.685 (χ^2^)	45/33
Survival ^c^	mean (95% CI)	19.8 (9.9–29.6)	16.8 (9.5–24.1)	25.5 (−1.3–52.2)	9.8 (3.5–16.1)	*p* = 0.591 (ANOVA)	18.1 (12.5–23.8)
	median (min–max)	9.3 (0.0–146.5)	11.9 (0.1–50.2)	17.3 (1.0–88.0)	10.5 (0.2–24.0)		10.9 (0.0–146.5)

^a^ growth pattern: m = mass forming; p = periductal; i = intraductal; ^b^ 0 = negative, 1 = positive; ^c^ months; (highly) significantly different (* *p* < 0.05, ** *p* < 0.01); ^#^ significantly different, *p* = 0.012 (ANOVA, Bonferroni *post hoc* test).

**Table 2:** Cox regression analysis for overall survival.

Variable		Groups or Cut-Off Values	Overall Survival			
			Method	*HR*	95% CI	*p*-Value
Clinicopathological characteristics	age	years	Enter ^1^	1.019	0.975–1.065	0.410
	Sex/Gender	groups male, female	Enter ^1^	0.797	0.344–1.844	0.596
	Localization	groups ih, ph, eh, gb	Enter ^1^	1.029	0.542–1.953	0.931
	Growth pattern	groups m, p, i	Enter ^1^	0.741	0.209–2.627	0.643
	Tumor size	cm	Enter ^1^	0.915	0.789–1.061	0.238
	pT ^1^	groups 1–4	Enter ^1^	0.771	0.245–2.422	0.656
	pN ^1^	groups N0, N1	Enter ^1^	0.489	0.108–2.207	0.352
	pM ^1^	groups M0, M1	Enter ^1^	3.683	0.998–13.588	0.050 *
			BW/Wald ^2^	2.999	1.355–6.636	0.007 **
	UICC ^1^	groups 1–4	Enter ^1^	1.729	0.340–8.780	0.509
	Grading ^1^	groups 1–4	Enter ^1^	0.945	0.344–2.598	0.913
MicroRNAs	−141 ^1^	low < 0.090 < high	Enter ^1^	0.592	0.175–2.001	0.399
	−200a ^1^	low < 0.104 < high	Enter ^1^	0.312	0.091–1.069	0.064
	−200b ^1^	low < 0.302 < high	Enter ^1^	0.392	0.121–1.272	0.119
			BW/Wald ^2^	0.376	0.180–0.786	0.009 **
	−200c ^1^	low < 0.343 < high	Enter ^1^	1.323	0.354–4.942	0.677
	−205 ^1^	low < 0.020 < high	Enter ^1^	3.396	1.007–11.448	0.049 *
			BW/Wald ^2^	2.436	1.083–5.482	0.031 *
	−429 ^1^	low < 0.020 < high	Enter ^1^	2.011	0.557–7.253	0.286
IHC	Vimentin ^1^	low < 36.25 < high	Enter ^1^	1.141	0.338–3.854	0.832
	*E*-Cadherin ^1^	low < 116.25 < high	Enter ^1^	0.829	0.288–2.388	0.728

^1^ Cox regression model: enter method; ^2^ Cox regression model: backward elimination Wald method; (highly) significantly different (* *p* < 0.05, ** *p* < 0.01). Abbreviations: BW, backward; eh, extrahepatic; gb, gall bladder; *HR*, hazard ratio; i, intraductal; ih, intrahepatic; IHC, immunohistochemistry; m, mass-forming; p, periductal; ph, perihilar; pM, distant metastasis status; pN, regional lymph node status; pT, primary tumor status; UICC, Union for International Cancer Control.

## Abbreviations

BTC	Biliary Tract Cancer
BMI1	BMI1 Proto-Oncogene, Polycomb Ring Finger
CD133	Prominin 1
CDH6	Cadherin 6
EMT	Epithelial to Mesenchymal Transition
FFPE	Formalin-Fixed Paraffin-Embedded
miR	MicroRNA
p14	Cyclin-Dependent Kinase Inhibitor 2A, Splice Variant
p16	Cyclin-Dependent Kinase Inhibitor 2A, Splice Variant
PCR	Polymerase Chain Reaction
PRC 1, 2	Polycomb Repressive Complex 1, 2
ROC	Receiver Operating Characteristic
SUZ12	SUZ12 Polycomb Repressive Complex 2 Subunit
ZEB 1, 2	Zinc Finger E-Box Binding Homeobox 1, 2
